# Relationship between serum soluble suppression of tumorigenicity (ST) 2 and global longitudinal strain in early onset preeclampsia

**DOI:** 10.1186/s12872-023-03696-9

**Published:** 2024-01-16

**Authors:** Hawani Sasmaya Prameswari, Cut Azlina Effendi, Achmad Fitrah Khalid, Setyorini Irianti, Ita Fatati, Mohammad Rizki Akbar

**Affiliations:** 1 Department of Cardiology and Vascular Medicine, Hasan Sadikin Central General Hospital, Universitas Padjadjaran, Bandung, West Java Indonesia; 2 Department of Obsetrics and Gynecology, Hasan Sadikin Central General Hospital, Universitas Padjadjaran, Bandung, West Java Indonesia; 3 Department of Obsetrics and Gynecology, Kiwari Regional General Hospital, Bandung, West Java Indonesia

**Keywords:** Global longitudinal strain, Preeclampsia, Soluble ST2

## Abstract

**Background:**

Preeclampsia is one of the leading causes of death in childbearing women worldwide. Hemodynamic changes in preeclampsia can trigger cardiac remodeling as indicated by increase of soluble-ST2 (sST2). Global longitudinal strain were able to detect systolic dysfunction better than the ejection fraction. This study aims to evaluate the correlation between serum levels of sST2 towards GLS in patients with early-onset preeclampsia.

**Methods:**

This is a cross-sectional observational study with correlation analysis. Subjects were patients with severe preeclampsia with gestational age before 34 weeks at Dr. Hasan Sadikin Central General Hospital Bandung and Bandung Kiwari Regional General Hospital from June to August 2022. Examination of sST2 was carried out through blood samples using the ELISA method. sST2 was measured using Presage ST2 Assay reagent. GLS examination was carried out using speckle tracking technique with EchoPAC. Correlation analysis was conducted using the Pearson test if normally distributed, otherwise Spearman’s correlation was conducted. Correlation analysis was followed by linear regression.

**Results:**

A total of 30 patients met the inclusion criteria. The mean age was 30.83 ± 7.09, with 17 (56.7%) multiparous patients. The median sST2 was 145.75 ng/mL, and the median GLS was − 17.4%. Spearman correlation analysis showed that there was a significant positive correlation with moderate strength between sST2 and GLS (r = 0.583; *p* < 0.002). Linear regression showed that every 1 ng/ml increase in sST2 would give an increase in GLS of 0.014%.

**Conclusion:**

There is a significant correlation between sST2 and GLS in patients with early onset severe preeclampsia.

**Supplementary Information:**

The online version contains supplementary material available at 10.1186/s12872-023-03696-9.

## Key message

• Subclinical myocardial dysfunction has been hypothesized as an underlying pathophysiology of subsequent cardiovascular event in preeclampsia patients.

• Patients with early onset preeclampsia have a higher baseline sST2 due to higher cytokine concentration.

• Higher sST2 likely to have worse GLS value despite normal LVEF.

• sST2 may be used to in daily practice to represent subclinical myocardial dysfunction in preeclampsia.

**Supplementary Information:**

The online version contains supplementary material available at 10.1186/s12872-023-03696-9.

## Introduction

Preeclampsia is one of the leading causes of death in childbearing women worldwide, causing 50.000 deaths every year [1]. The incidence of preeclampsia in Indonesia was 5.3% or 128.273 per year [2]. Preeclampsia was defined as a clinical syndrome including new onset hypertension that occur in second half of pregnancy. The proposed mechanism of preeclampsia includes abnormal trophoblastic invasion, maternal immunological factor, inflammation and genetic. Abnormal placement of placenta would also lead to release of angiogenic factor giving rise to endothelial dysfunction and systemic vasospasm. Patients with history of preeclampsia had been shown to have an increased risk of myocardial infarction, heart failure, and stroke [3–6].

Preeclampsia could lead to left ventricular remodelling as an adaptive response to preserved stroke volume, which also increase myocardial stress [3]. Those with history of preeclampsia have a 2 times higher risk of developing cardiovascular events and those with early onset preeclampsia, defined as preeclampsia that occurs before 34th week, will have up to 7 times higher risk of cardiovascular events in the future [3, 7]. Subclinical cardiac dysfunction in preeclampsia were thought to be the underlying pathophysiology in which subsequent cardiovascular event occur. Strain echocardiography assessment using Global Longitudinal Strain (GLS) could identify subclinical cardiac dysfunction with high accuracy and also describes global and segmental LV function. Several studies has shown worse GLS in preeclampsia and return to normal after delivery [8–10].

Biomarker also plays an important role in myocardial injury, fibrosis and cardiac remodelling. High-sensitivity soluble Suppression of Tumorigenicity-2 (sST2) assays have been developed and used as valuable adjuncts for prognosis and monitoring of Heart Failure (HF) and were included in the 2017 American College of Cardiology/American Heart Association update of heart failure guidelines [11]. Soluble ST2 was found to play an important role in cardiovascular disease, not only as a predictor of hospitalization and death but also as a factor in determining the prognosis of heart failure patients [12, 13]. Endothelial dysfunction, placental secretion, ventricular hypertrophy, and remodeling have all been linked with elevated levels of sST2 in patients with preeclampsia [14–16]. Based on this findings, recognizing early left ventricular remodeling is crucial in managing preeclampsia patient. Therefore, this study was aimed to examine the relationship between GLS and sST2 in early onset preeclampsia as this population was at higher risk of cardiovascular events.

## Materials and methods

### Subject recruitment and ethics statement

This study was approved by the West Java Research Ethics Committee, and written informed consent was obtained from each participant. All the methods included in this study are in accordance with the declaration of Helsinki. Patients considered eligible for this study met the following criteria: the patient (1) must be diagnosed with early onset preeclampsia (< 34 weeks) by a certified obstetrician; (2) must have a history of at least one visit to the designated hospital (Dr. Hasan Sadikin Central General Hospital and Kiwari Regional General Hospital Bandung); (3) must have no history or signs of valvular heart disease, congenital heart disease, myocarditis, left ventricular ejection fraction < 50%, asthma, sepsis, malignancy, obesity, diabetes mellitus and autoimmune disease. Pre-eclampsia was defined as the new onset of a systolic blood pressure (BP) ≥ 140 mmHg or diastolic blood pressure ≥ 90 mmHg after 20th week of gestation accompanied with new onset proteinuria ≥ 300 mg in a 24 h urine collection, 50 mg/mmol protein/creatinine ratio or at least 2 + on dipstick testing on two consecutive measurements [3] We included all subjects that meet the inclusion and exclusion criteria, a total of 30 patients were included after applying the exclusion criteria.

### Sample collection and detection of sST2 by ELISA

Blood samples were collected from the cubital vein of the left arm by an expert nurse, 3 ml of blood were taken into tubes containing Ethylene diamine tetra-acetic acid (EDTA). The samples then stored and transported to the Department of Clinical Pathology of RSUP Dr. Hasan Sadikin Bandung within 30 min with a cooler box at 40 C and were centrifuged for 15 min at 4^o^C and stored at 2-8^o^C. Maternal plasma of sST2 was measured using the Presage® ST2 Assay according to the manufacturer’s instruction.

### Global longitudinal strain measurement

Detailed echocardiographic evaluation was performed at admission. The examination was done using Vivid 7 (GE Vingmed Ultrasound AS, Horten, Norway). Echocardiographic calculations of chamber quantification were performed in accordance with the recommendations of The American Society of Echocardiography 2015 [17]. Cardiac images were scanned at long-axis apical three chambers, two chambers, and four chambers view. The mean frame rate was 60 frames per second (range 50–70). Data was stored on the hard disc of the echocardiographic machine, and transferred to a workstation (EchoPAC PC, GE Vingmed) for offline analysis. The system calculates mean global strain and strain rate values for all predetermined LV segments. All echocardiography images and strain values measurement were performed by one cardiologist specialized in echocardiography to minimize variability.

### Statistical analysis

The Saphiro-Wilk test was used to assess the distribution of the data. Normally distributed data will be analyzed using Pearson test and for those whose data not normally distributed, we used the Spearman test to correlate variables between the groups. Correlation coefficient was deemed very low, low, moderate, strong, very strong according to r value (0–0.199. 0.2–0.399, 0.4–0.599, 0.6–0.799, 0.8–1.0 respectively) with *p* value < 0.05 was considered as statistically significant. Variables at antepartum period will be compared with postpartum period using paired t-test when data was normally distributed Wilcoxon test will be used when data was non-normally distributed. Statistical analyses were performed with STATA, version 24.0 (STATA Corp LCC, Texas, USA).

## Results

This study included a total of 30 subjects. The participants had a mean age of 30.83 ± 7.09 years with median gestational age of 31 weeks. There were similar results in terms of parity between primiparity and multiparity. Echocardiography parameters showed slightly elevated LVMI at median of 112.91 g/m^2^, normal size of LA based on LAVI and mild diastolic dysfunction was found at 20 participants (66.7%). All participants had methyldopa as medication for high blood pressure. Mean soluble ST2 was within normal range 145.75 ng/ml and GLS was found slightly decrease at 17.4% (based on non-pregnant reference). The characteristics of this study population was listed in Table 1.

**Table 1:** Baseline characteristics

Variables	Total n = 30
Age (years)*	30.83 ± 7.09
Gestational age (weeks)**	31 (30–32)
Parity, n (%)	
Primipara	13 (43.3)
Multipara	17 (56.7)
BMI, kg/m^2^	
Before pregnancy*	21.2 (20.58–21.63)
Present state*	28.85 (26.2-29.35)
Weight gain during pregnancy*	9.77 ± 1.43
Blood pressure (mmHg)**	
Systolic	155 (147–160)
Diastolic	90 (90–100)
Echocardiography parameters	
LVMI, g/m^2^*	112.91 ± 24.78
LAVI, mL/m^2^*	23.39 ± 4.72
LVEF, %*	60.13 ± 6.56
Diastolic dysfunction	20 (66.7)
Normal	10 (33.33%)
Grade I	20 (66.67%)
Metildopa 500 mg, n(%) sST2 (ng/mL) GLS (%)	30 (100) 145.75 (37.5–297.5) -17.4 (-13,3 – (-20,1))

BMI: Body mass index, LAVI: Left atrial volume index, LVEF : Left ventricular ejection fraction, LVMI : Left ventricular mass index, sST2: soluble suppression of tumorigenicity-2, GLS: Global longitudinal strain, SD: standard deviation

*Data was shown as mean (±SD)

**Data was shown as median (minimum-maximum)

Bivariate analysis found that systolic blood pressure (r 0.534, p 0.002) and left ventricular ejection fraction (r -0.386, p 0.035) was correlated with sST2, while sST2 itself was significantly correlated with GLS (r 0.583, p 002). Linear regression analysis showed that every elevation of sST2 by 1 ng/ml will increase GLS value by 0.014%. All analysis was listed in Table 2.

**Table 2:** Correlation Between Variables and sST2

Variables	*P* value
Age (years)	NS
Gestational age	NS
Parity	NS
Body mass index prior before pregnancy	NS
Current body mass index	NS
Systolic blood pressure	0,002
Diastolic blood pressure	NS
LV mass index	NS
LAVI	NS
LV ejection fraction	0,035
Diastolic dysfunction	NS

## Discussion

Risk of cardiovascular events in preeclampsia patients remain high even after pregnancy had been terminated or complete recovery of the blood pressure, this phenomenon could be caused by severe myocardial and endothelial injury [4, 18]. The degree of myocardial injury depends on the severity and onset of preeclampsia. Soluble ST2, which is a marker of fibrosis, will be elevated proportionately with severity of preeclampsia [19]. Our previous study found that median sST2 of third trimester pregnancy were 66.48 ng/ml and it was found that preeclampsia was the most contributing factor in the elevation of sST2 in pregnancy [20]. Stampalija et al. found that median value of sST2 was 76.1 ng/ml and the value of sST2 is higher depends on the severity of preeclampsia [21]. Maharani et al. also found that sST2 is significantly elevated in preeclampsia population and tend to increase in preeclampsia with complication [19].

Mean value of sST2 in this study is 145.75 ng/ml, which is higher than other similar studies. This wide difference of median sST2 may have been caused by a disproportionate occurrence of early- and late-onset preeclampsia as most of previous studies do not differentiate the population based on onset of preeclampsia. Women with early-onset preeclampsia had higher sST2 concentrations than those with late-onset preeclampsia [7, 21]. This phenomenon resulted from higher cytokine concentrations (such as IL-12, TNF-α, and IL-1β) in early- versus late-onset preeclampsia [15]. Studies also showed that women with history of early preeclampsia, which occur before 34th week of gestational age, has higher risk up to 7 times of developing cardiovascular event [22]. This mechanism occurs as early onset preeclampsia has more severe placental damage compared with late onset preeclampsia. Prolonged ischemic condition of placenta play a significant role in the pathogenesis of preeclampsia [21].

Global longitudinal strain is an useful tool to detect subclinical myocardial dysfunction. Median GLS value of this study was − 17,4% (-13,3 – (-20,1%)). Our previous study found that mean GLS − 16,6 ± 1,1% based on observational study of preeclampsia patients in Hasan Sadikin General Hospital [20]. Similar result was also found in study by Akbar et al. in which mean GLS value was − 16.2%.^8^ Biochemical changes and high blood pressure will increase myocardial afterload. These changes will increase oxygen demand and microvascular ischemia will occur and causing myocardial fibrosis [23]. This study showed that subclinical myocardial dysfunction occur in majority of study sample despite normal LVEF. This was major limitation in detection of myocardial dysfunction as LVEF only saw geometrical changes.

Soluble ST2 was found correlated with GLS (r 0.583, p 0.002) in this study. This finding was similar with other studies. Study conducted by Akbar et al. showed moderate positive correlation between sST2 with GLS value (r 0.439, p 0.015) [8]. Aula et al. also found reduction of GLS value in patients with high sST2 in cancer patients treated with chemotherapy and stated that chemotherapy was the most contributing factor in GLS reduction (p 0.034) [24]. This study also found that every sST2 elevation by 1 ng/ml will increase GLS value by 0.014%. Similar result was found in our previous study, even though the relation between sST2 and GLS was weaker in this study. This could be caused by range of sST2 and GLS value in this study was wider than our previous study ((37,5–297,5 ng/ml and 51,75–85,42 ng/ml for sST2;-13,3 – (-20,1%) and (-8,6 – (-21,6%), respectively) [8]. Based on these findings, higher sST2 likely to have worse GLS value despite normal LVEF in early onset preeclampsia patients due to longer exposure of inflammatory substrates. This inflammatory process could lead to elevation of sST2 levels that acts as a decoy receptor, which could lead to apoptosis and fibrosis [25]. Reduction of LVEF may not occur early as this inflammatory process tends to decrease after delivery, however subclinical myocardial dysfunction caused by this process could be harmful in the future. Higher GLS, which indicating a subclinical myocardial dysfunction, has also been linked with higher future cardiovascular events [26–28]. Based on this findings, soluble ST2 may be able to represent subclinical myocardial dysfunction in pregnancy. However, sST2 level varies based on severity of preeclampsia and its ability to predict recovery of GLS in post-partum period remains unknown.

Diastolic dysfunction was found in 20 patients (66.7%). Even though the result was statistically non-significant, maternal diastolic dysfunction was common findings in patients with preeclampsia. Study showed that impaired diastolic function may occur before and alongside the onset preeclampsia [29–31]. Maternal physiological cardiovascular changes were maladapted in pregnant patients with hypertension. This maladaptations also strongly correlated with pregnancy outcome. Impairment of diastolic function of left ventricle precedes systolic dysfunction and might be an indicator to predict the risk of pregnancy complications [31]. Study by Muthyala also showed that diastolic dysfunction was occurred in one-fifth of women with preeclampsia. Diastolic dysfunction was also correlated with the severity of preeclampsia. It was showed that diastolic dysfunction was more common found in severe preeclampsia (39%) compared with mild preeclampsia (3.3%).^30^

### Limitations

This study has several limitations. This study do not differentiate degree of preeclampsia, as sST2 levels was found higher in those with severe preeclampsia. Lack of control group in this study may limit the accuracy of this study findings. All of our samples were consuming anti-hypertensive therapy, which may impact the result of this study.

## Conclusion

There was a positive correlation between sST2 and GLS value in early onset preeclampsia. Soluble ST2 may be used to in daily practice to represent subclinical myocardial dysfunction in preeclampsia as patients with subclinical myocardial dysfunction have higher cardiovascular events in the future.

### Electronic supplementary material

Below is the link to the electronic supplementary material.

Supplementary Material 1

## Data Availability

All data regarding this study are open for public. Please contact Hawani Sasmaya Prameswari (hawanisasmaya@gmail.com) for data sharing information.
